# Acutalibacter caecimuris sp. nov., Acutalibacter intestini sp. nov. and Neglectibacter caecimuris sp. nov., three novel species of the family Oscillospiraceae isolated from caecal contents of C57BL/6J mice

**DOI:** 10.1099/ijsem.0.006449

**Published:** 2024-07-12

**Authors:** Jia-Hui He, Chang-Yu Wang, Rashidin Abdugheni, Xue Ni, Chang Liu, Ming-Xia Bi, Shuang-Jiang Liu

**Affiliations:** 1 College of Veterinary Medicine, Shanxi Agricultural University (Shanxi Academy of Agricultural Sciences), Jinzhong 030801, PR China; 2 State Key Laboratory of Microbial Resources, Institute of Microbiology, Chinese Academy of Sciences, Beijing 100101, PR China; 3 School of Life Sciences, Division of Life Sciences and Medicine, University of Science and Technology of China, Hefei 230001, PR China; 4 State Key Laboratory of Desert and Oasis Ecology, Key Laboratory of Ecological Safety and Sustainable Development in Arid Lands, Xinjiang Institute of Ecology and Geography, Chinese Academy of Sciences, Urumqi 830011, PR China; 5 State Key Laboratory of Microbial Technology, Shandong University, Qingdao 266237, PR China

**Keywords:** *Acutalibacter caecimuris*, *Acutalibacter intestini*, mouse gut microbime, *Neglectibacter caecimuris*, *Oscillospiraceae*

## Abstract

The intestines of mice are colonized by diverse, as-yet-uncultivated bacteria. In this report, we describe the isolation, culture, genotypic and phenotypic characterization, as well as taxonomic classification of three novel anaerobic bacterial strains derived from the caecal contents of C57BL/6J male mice. According to the phenotypic and genotype-based polyphasic taxonomy, we propose three novel species within the family *Oscillospiraceae*. They are *Acutalibacter caecimuris* sp. nov. (type strain M00118^T^=CGMCC 1.18042^T^=KCTC 25739^T^), *Acutalibacter intestini* sp. nov. (type strain M00204^T^=CGMCC 1.18044^T^=KCTC 25741^T^) and *Neglectibacter caecimuris* sp. nov. (type strain M00184^T^=CGMCC 1.18043^T^=KCTC 25740^T^).

## Introduction

Mouse models have a pivotal role in the study of host–microbe interactions, and host-associated microbes regulate host health and diseases. In particular, the C57BL/6J mouse has been widely used in studies of host health and disease. A lack of cultivated gut microbes originating from the mouse limits the study of host–microbe interactions. In 2016, Lagkouvardos and colleagues established the mouse intestinal bacterial collection (miBC) containing 76 species (www.dsmz.de/miBC) [1], 74 % of which belong to the phylum *Firmicutes*. Later, in 2020, Liu *et al*. constructed the mouse gut microbial biobank (mGMB, https://cgmcc.net/english/mgmb) [2], which increased the cultivated mouse gut bacterial species to 180. *Firmicutes* was still the dominant phylum in the mouse gut. Among the members of the phylum *Firmicutes*, the families *Lachnospiraceae* and *Oscillospiraceae* were the most abundant bacterial taxa and the major butyrate-producers [3,6]. The abundance of the *Oscillospiraceae* members was reduced in malnourished mice [7], suggesting that members of the family *Oscillospiraceae* might be correlated with host nutirition and health.

Currently, the family *Oscillospiraceae* contains 66 validly published and nominated genera (https://lpsn.dsmz.de/family/oscillospiraceaeFamily: Oscillospiraceae (dsmz.de)). Among the genera, the genera *Acutalibacter* (https://lpsn.dsmz.de/genus/acutalibacter)Genus: Acutalibacter (dsmz.de) and *Neglectibacter* (https://lpsn.dsmz.de/genus/neglectibacter)genus: Neglectibacter (dsmz.de) are each represented by a single species. The type strains of the type species are *Acutalibacter muris*Acutalibacter muris KB18^T^ (=DSM 26090^T^=KCTC 15540^T^), isolated from mouse faeces [1], and *Neglectibacter timonensis* SN17^T^Neglectibacter timonensis SN17T (=CSUR P2265^T^=DSM 102082^T^), isolated from human faeces [8]. Both A. muris*A. muris* and *N. timonensis* were reported to be Gram-stain-positive, strictly anaerobic, oxidase-negative and catalase-negative [1,8]. Their major cellular fatty acids were C_16 : 0_, anteiso-C_15 : 0_ and iso-C_15 : 0_ [1,8].

Here, we report the isolation, genotypic and phenotypic characterization, and polyphasic taxonomy of three strains, namely M00118^T^, M00204^T^ and M00184^T^, from the caecal contents of C57BL/6J mice. Our results showed that strains M00118^T^ and M00204^T^ represented two novel species of the genus *Acutalibacter* and that strain M00184^T^ represented a novel species of the genus *Neglectibacter*, of the family *Oscillospiraceae*.

## Methods

### Sample collection and treatment

All samples were obtained from the caecal contents of specific-pathogen-free (SPF) male C57BL/6J mice. The animal ethics application number is SYDWLL-2022-022, approved by the State Key Laboratory of Microbiology, Shandong University. Fresh mouse caecal contents were transferred immediately after collection to an anaerobic workstation (AW 500SG, Electrotek) with a nitrogen content of 85 % N_2_, 5 % CO_2_ and 10 % H_2_ [9], fresh samples were dissolved in 1×PBS solution (pH 7.2–7.4; P1022, Solarbio) and repeatedly pipetted to obtain a suspension. The suspension was then filtered through a 40 µm cell sieve (Biosharp) and serially diluted with PBS solution, spread on modified GAM (mGAM; HB8518, Hopebio) agar plates, and incubated anaerobically at 37 °C [10].

### Culture media, bacterial isolation, and cultivation

mGAM broth was used for bacterial isolation and cultivation. mGAM (1 l) was supplemented with 0.5 g l-cysteine hydrochloride (C14772120, Macklin), 0.5 g l-arginine (H2113165, Aladdin), 0.3 g l-tryptophan (C11677452, Macklin), 2.0 g NaHCO_3_ (10 018 960, Sinopharmgroup), 2.46 g CH_3_COONa (C15676262, Macklin), 5 ml haemoglobin chloride (C11677452, Macklin), 1 ml resazurin (C11677452, Macklin), 100 ml clarified rumen fluid (A110101, Wizbiotech), 50 ml goat blood (HQ60071, HongQuan Bio), 5 ml vitamin K1 solution (HB8462, Hopebio), 1 ml Wolfe’s vitamin solution (SL0110, Coolaber) and 1 ml Wolfe’s mineral solution (SL0120, Coolaber) with d-mannose (C14246372, Macklin), d-fructose (C12015855, Macklin), palatinose hydrate (E2025150, Aladdin), inulin (C15492472, Macklin), d-galactose (C15131463, Macklin), palatinose (C14786871, Macklin), l-rhamnose (C15884351, Macklin), cellobiose (B2311508, Aladdin) and trehalose (C14894221, Macklin), all at 0.5 g l^−1^, and the pH was adjusted to 7.2. The treated dilutions were spread on mGAM agar plates. The inoculated agar plates were incubated at 37 °C under anaerobic conditions. Colonies were picked, streaked and cultivated on mGAM agar and identified by 16S rRNA gene sequencing to ensure purity. All operations were performed in an anaerobic workstation (D500G, GeneScience) with a nitrogen content of 85 % N_2_, 5 % CO_2_ and 10 % H_2_.

### Cell morphology observation, and chemotaxonomic analysis

Bacterial cells were cultivated under anaerobic conditions at 37 °C for 3–7 days, and the cellular morphology was observed with transmission electron microscopy (JEM-1400, jeol). Gram-staining was performed using a Gram-stain Kit (G1060, Solarbio) and spores were stained using a kit (G1132, Solarbio), according to the manufacturer’s instructions. Gram-staining and spore-formation were observed with light microscopy (Eclipse Ts2R, Nikon). Oxygen requirement was determined in liquid mGAM medium without cysteine. Bacteria were inoculated into glass tubes, sealed with rubber stoppers, and incubated at 180 r.p.m. on a shaker (ZQZY-CFSW, Zhichu) at 37 °C. The strains were grown in the liquid mGAM medium at different temperatures (16, 20, 25, 30, 35, 37, 45, 50 and 65 °C) to evaluate temperature tolerance and optimum growth under an anaerobic atmosphere. Growth of the strains at different pH values (pH 4.0, 5. 0, 5.5, 6.0, 6.5, 7.0, 7.5, 8.0 and 9.0) and NaCl concentrations (0, 0.5, 1.0, 1.5, 2.0, 2.5, and 3.0 % w/v) was conducted under anaerobic conditions at 37 °C. All cell growth was measured as OD_600_ using a UV/visible spectrophotometer (Harvard Biochrom Ultrospec 10) and all experiments were conducted in triplicate. The disc diffusion method [11] was used to evaluate resistance to antibiotics (Bikeman). The tested antibiotics were (per disc) 10 µg kanamycin, 5 µg cefixime, 30 µg chloramphenicol, 5 µg rifampin, 10 µg penicillin, 10 µg carbenicillin, 10 µg ampicillin, 2 µg clindamycin, 25 µg amoxicillin, 30 µg cefoperazone, 10 µg gentamicin, 30 µg tetracycline, 5 µg ciprofloxacin, 300 µg polymyxin B, 15 µg azithromycin, 10 µg streptomycin, 0.04 µg bacitracin, 30 µg vancomycin, 15 µg clarithromycin and 15 µg erythromycin. The inhibition zone diameters of strains M00118^T^ and M00184^T^ were measured after 3 days of incubation on mGAM agar plates, while strain M00204^T^ was measured after 7 days of incubation. Enzymatic activity on different substrates after 4 h of incubation at 37 °C was tested using the API ZYM kit (bioMérieux). Twenty acid production reactions and catalase activity in anaerobes over 48 h at 37 °C were identified using the API 20A Anaerobe Test Kit (bioMérieux). Immediate bubble formation was observed for oxidase- and catalase-positive strains when two drops of 30 % H_2_O_2_ were added. AN MicroPlates (1007, Biolog) were used to detect the utilization of 95 carbon sources by three strains [12]. Strains cultivated in mGAM liquid medium were utilized to harvest cells for the tests, which were conducted in compliance with the manufacturer’s instructions. By following previously described procedures, new bacteria were anaerobically fermented in mGAM liquid medium at 37 °C for 3–7 days to assess the amount of cellular fatty acids, respiratory quinones, and polar lipids [10,13, 14].

### 16S rRNA gene sequencing and phylogenetic analysis

For the phylogenetic analysis of M00118^T^, M00204^T^ and M00184^T^, the 16S rRNA genes of the three bacterial strains were amplified using the universal primers 27F (5′-GAGAGTTTGATCCTGGCTCAG-3′) and 1492R (5′-TACGGYTACCTTGTTACGACTT-3′) [14]. Each 30 µl reaction mixture contained 1 µl (10 mM) of each primer, 15 µl PCR Master Mix (CWBIO), 3 µl template DNA, and 10 µl ddH_2_O. The PCR programme consisted of heating at 94 °C for 4 min, followed by 30 cycles of 94 °C for 0.5 min, 55 °C for 0.5 min, and 72 °C for 0.75 min, and an extension at 72 °C for 5 min. Sequencing was performed by Beijing Tsingke Biotech Co., Ltd. (Qingdao, PR China) using PCR products. The obtained 16S rRNA gene sequences were aligned with the NCBI database (https://www.ncbi.nlm.nih.gov/) [15] using blast (https://blast.ncbi.nlm.nih.gov/). Subsequently, 16S rRNA gene sequences of validly published type strains of the genus *Acutalibacter* and *Neglectibacter* were downloaded from the List of Prokaryotic Names with Standing in Nomenclature database (http://www.bacterio.net) [16]. Phylogenetic analysis of 16S rRNA gene sequences was performed using mega-X [8,17]. The neighbour-joining (NJ) method [18] and Kimura’s two-parameter model [19] with 1000 bootstrap replicates [20] were used to reconstruct phylogenetic trees. *Bacillus subtilis* IAM 12118^T^ was used as the outgroup. In addition, the maximum-likelihood and maximum-parsimony methods [21,22] were used to reconstruct phylogenetic trees (Fig. S3, available in the online Supplementary Material) to verify the validity of the NJ tree.

### Genome sequencing and genome-based analysis

Cells grown in mGAM broth were harvested by centrifugation and genomic DNA was extracted using the QIAamp DNA Mini Kit (51 306, Qiagen) following the manufacturer’s instructions. Genomes were sequenced using the HiSeq X-Ten platform (Illumina), clean reads were obtained, and all genomes were assembled and annotated using Glimmer3 software and coding gene predictions. Regarding ncRNA genes (mainly tRNA genes and rRNA genes), predictions were made using tRNAscan-SE and rRNAmmer software. Phage Finder software was used to predict prophages in bacterial whole genomes. In addition, genomic islands were predicted for all strains using IslandPath-DIOMB. CRISPR prediction for all genomes was performed using CRISPRdigger. kegg annotation of the genomes was also carried out. The phylogenomic tree was reconstructed using CVTree 4.0 (CVTree 4.0http://cvtree.online/v4/prok/index.html) [23]. Phylogenomic trees based on whole genomes were reconstructed using the CVTree method and the NJ method [23]. The CVTree method is an alignment-free method based on K-tuple counting and background subtraction, also known as the composition vector method [24,25]. It included the genome sequences of the three strains and their corresponding phylogenetic neighbours, validly published strains whose 16S rRNA gene sequence similarities to the three type strains ranged from 90.05 to 97.10 %, and included *Bacillus subtilis* IAM 12118^T^ as an outgroup strain. The average nucleotide identity (ANI) values [26,27] were calculated using the Orthologous Average Nucleotide Identity Tool (OAT) version 0.93.1 and an unweighted arithmetic mean dendrogram (UPGMA) was generated based on the ANI values. Digital DNA–DNA hybridization (dDDH) [28] values were calculated using the online Genome-to-Genome Distance Calculator (version 2.1; https://ggdc.dsmz.de/ggdc.php) [29]. The dDDH values and the ANI scores were based on the three strains and their phylogenetic neighbours.

### Culture preservation

Pure bacteria samples cultivated in mGAM liquid medium (500 µl) were added to 30 % (v/v) glycerol (500 µl) and stored at −80 °C for laboratory-scale preservation. All type strains were deposited at the China General Microbiological Culture Collection Center (CGMCC) and the Korean Collection for Type Cultures (KCTC). Strain deposit numbers are given in the species description.

## Results and discussion

### Bacterial origin, growth and cell morphology

**Fig. 1.**
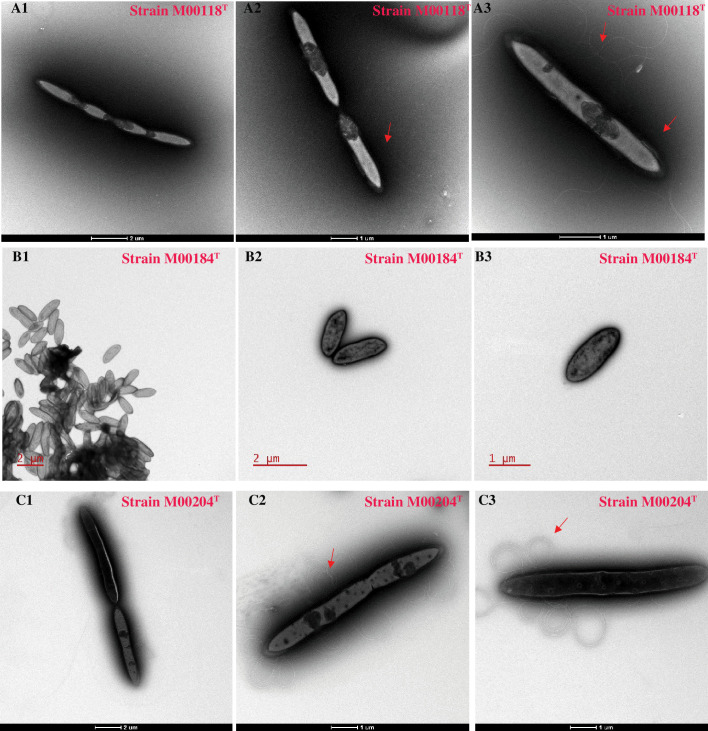
Transmission electron microscope images of strains M00118^T^ (**a1, a2, a3**), M00184^T^ (**b1, b2, b3**), and M00204^T^ (**c1, c2, c3**). The flagella of the bacterial strains are indicated with arrows.

Strains M00118^T^, M00204^T^ and M00184^T^ were isolated from caecal contents of SPF C57BL/6J mice and grew under anaerobic conditions on mGAM agar plates. They were Gram-positive, non-spore-forming and unable to grow under aerobic conditions. They formed visible single colonies when grown anaerobically on mGAM agar plates at 37 °C for 3–7 days. Colonies of strain M00118^T^ had a smooth, translucent, omelette-like surface with irregular edges and did not glisten, those of strain M00204^T^ were creamy white, smooth, opaque, raised with irregular edges and glossy, and those of strain M00184^T^ were white, smooth, opaque, rounded, raised, neatly edged and shiny (Fig. S1). Cells of strain M00118^T^ were rod-shaped with flagella (Fig. 1a1/a2/a3), those of strain M00204^T^ were rod-shaped with flagella (Fig. 1c1/c2/c3), and those of strain M00184^T^ were short rod-shaped without flagella (Fig. 1b1/b2/b3). Additional features of the strains are available in the species description.

### Cellular fatty acids and polar lipid profiling

The major cellular fatty acids (>5 %) of each strain were as follows: strain M00118^T^ had C_16 : 0_, anteiso-C_15 : 0_, iso-C_14 : 0_, iso-C_15 : 0_ and iso-C_16 : 0_; strain M00204^T^ had C_16 : 0_, C_16 : 1_ 2-OH, anteiso-C_15 : 0_, iso-C_15 : 0_ and iso-C_16 : 0_; and strain M00184^T^ had C_16 : 0_, anteiso-C_15 : 0_, iso-C_14 : 0_ and iso-C_15 : 0_. More detailed cellular fatty acid profiles are presented in Table S1. The polar lipid profiles of the three strains contained diphosphatidylglycerol (DPG), phosphatidylglycerol (PG), glycolipids (GL) and phospholipid (PL) (Fig. S2). The major quinone in all three strains was menaquinone 6 (MK-6).

### General features of genomes

The genomes of all strains were sequenced and assembled. The genomes of strains M00118^T^ and M00204^T^ were most similar to *A. muris* KB18^T^, and the genome of strain M00184^T^ was most similar to *N. timonensis* SN17^T^. The genome size of strain M00118^T^ was 3 476 835 bp, of strain M00204^T^ was 4 293 150 bp, and of strain M00184^T^ was 3 089 163 bp. General information about each genome is provided in Table 1. Their encoding capacities are described in the following paragraphs in terms of carbohydrate metabolism, amino acid metabolism, transports, cofactor and vitamin, signal transduction, nucleotide metabolism, and energy metabolism.

**Table 1.** Genomic differentiation on strains M00118^T^, M00204^T^, M00184^T^ and type strains of closely related species

Characteristic	*A. caecimuris*M00118^T^	*A. intestini*M00204^T^	*A. muris*KB18^T^ [1]	*N. caecimuris*M00184^T^	*N. timonensis*SN17^T^ [8]
Genome size (bp)	3 476 835	4 293 150	3 802 913	3 089 163	3 671 396
DNA G+C contents (mol%)	56.67	53.44	54.6	52.90	52.8
Contigs	101	158	1	21	14
Coding genes	3971	3018	3879	4437	3288
Coding gene size (bp)	301 611	2 592 339	3 372 380	3 450 027	3 224 862
Non-coding genes(tRNAs and rRNAs)	59	57	60	54	65
Prophages	3	2	na	1	na
Genomic islands	17	20	na	8	na
CRISPR	0	0	na	0	na

### The three bacterial strains represent three novel species in two genera

**Fig. 2.**
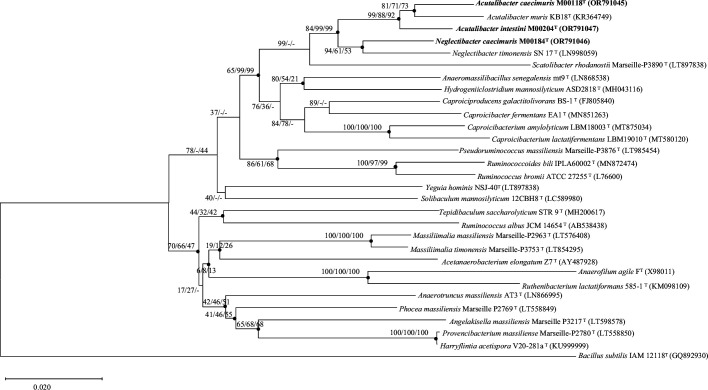
Phylogenetic tree of strains M00118^T^, M00204^T^, M00184^T^ and their closely related type strains. The tree is based on 16S rRNA gene sequences and generated using the neighbour-joining (NJ) method with Kimura’s two-parameter model. The maximum-likelihood (ML) and maximum-parsimony (MP) methods were utilized to reconstruct phylogenetic trees to verify the validity of the NJ tree. Filled circles indicate branch nodes that were also found in trees generated with the ML and MP methods. The numbers at branch nodes represent the percentage of associated taxa clustered together in the bootstrap test (1000 replicates; NJ/ML/MP). The novel species are shown in bold. *Bacillus subtilis* IAM 12118^T^ was considered as an outgroup. GenBank accession numbers are given in parentheses. Bar, 0.02 substitutions per site.

The 16S rRNA gene sequence identities of strains M00118^T^, M00184^T^ and M00204^T^ compared to validly published bacterial species were below the species identification threshold of 98.65 % [30,31]. Phylogenetic data showed that they were associated with the genera *Acutalibacter* and *Neglectibacter*, and strains M00118^T^ and M00204^T^ clustered with members of the genus *Acutalibacter* (Figs2  3). The dDDH values among strains M00118^T^, M00204^T^ and M00184^T^ and species of the *Acutalibacter* and *Neglectibacter* were below the species cutoff value of 70 % [32]. The ANI values (%) and the ANI-based UPGMA dendrogram trees are presented in Fig. 4. The ANI values of all species were below the species discrimination cut-off value of 95 % [33]. The 16S sequence similarity between strains M00118^T^ and M00204^T^ was 97.07 %. The ANI and dDDH values between their genomes were 76.78 and 26.90 %, respectively. Based on analyses of the 16S rRNA gene, as well as the genomic and phenotypic characterization, we concluded that strains M00118^T^ and M00204^T^ represent two novel species in the genus *Acutalibacter*, while strain M00184^T^ belongs to the genus *Neglectibacter*. Conclusive phenotypic results are detailed in the following paragraphs.

**Fig. 3.**
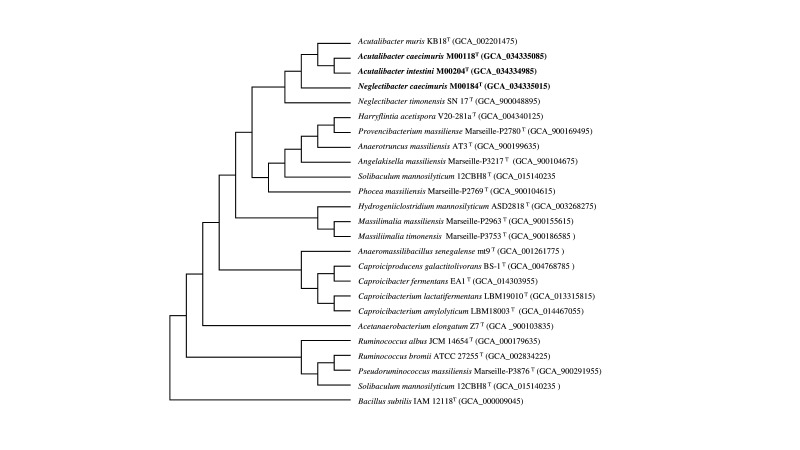
Phylogenomic tree of strains M00118^T^, M00204^T^, M00184^T^ and their phylogenetically closely related neighbours. The tree is based on whole genomes and reconstructed using CVTree. The novel species are shown in bold. *Bacillus subtilis* IAM 12118^T^ was used as an outgroup. GenBank accession numbers are shown in parentheses.

**Fig. 4.**
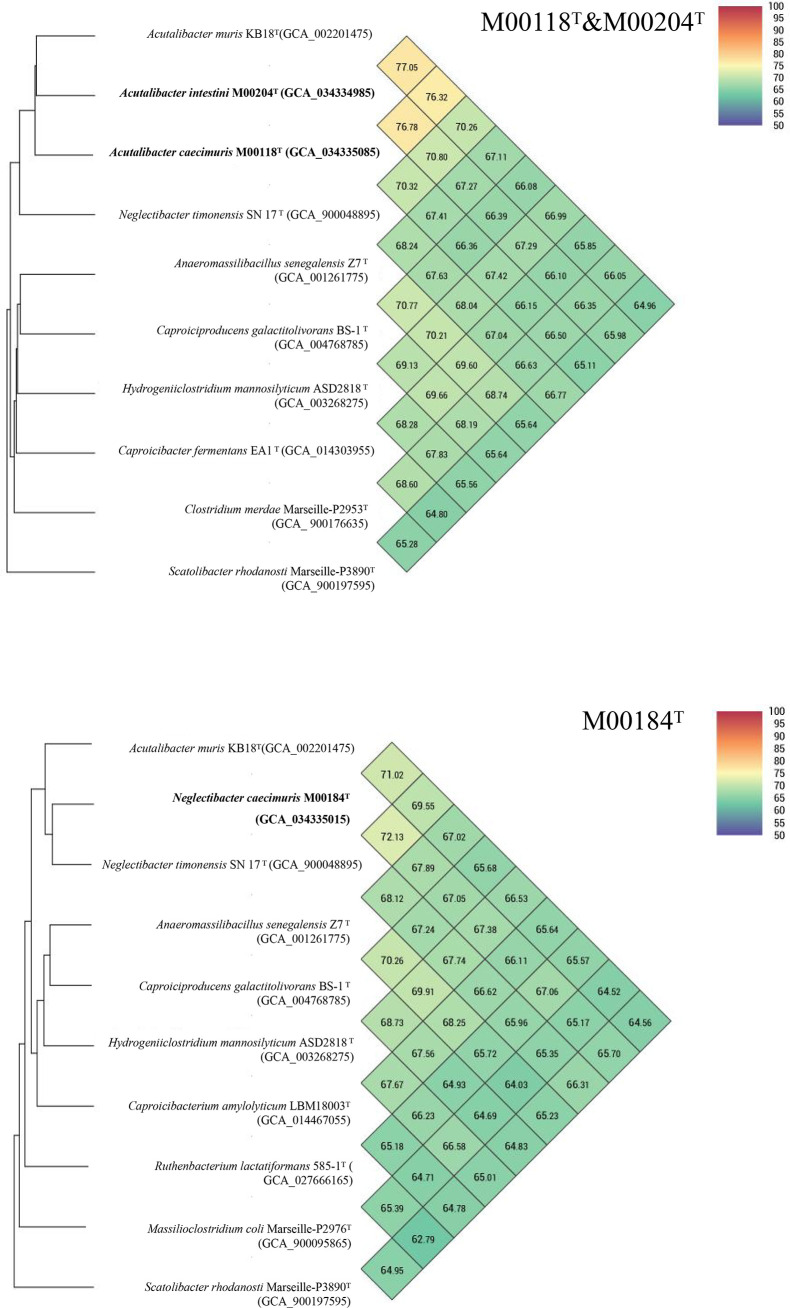
UPGMA phylogenetic trees and ANI heat maps based on whole genomes. The UPGMA phylogenetic trees and the ANI heat maps display the connections between strains M00118^T^, M00204^T^, M00184^T^ and their closely related neighbours. The novel species are shown in bold. GenBank accession numbers of the genomes are shown in parentheses.

#### Strain M00118^T^

Phylogenetic analyses indicated that strain M00118^T^ was clustered within the genus *Acutalibacter* of the family *Oscillospiraceae*. It was most closely related to *A. muris* KB18^T^ (97.10 % 16S rRNA gene sequence similarity), and had a G+C content of 56.67 mol%. kegg annotation of its genome revealed 133 genes related to carbohydrate metabolism, 113 genes related to amino acid metabolism, 78 genes related to cofactors and vitamins, 72 genes related to nucleotide metabolism, 67 genes related to energy metabolism, 36 genes related to lipid metabolism, 28 genes related to glycan biosynthesis and metabolism, 24 genes related to biosynthesis of other secondary metabolites, 15 genes related to terpenoids and polyketide metabolism, and 11 genes related to xenobiotic biodegradation and metabolism. The ANI and dDDH values between the genomes of strain M00118^T^ and *A. muris* KB18^T^ were 76.32 and 25.90 %, respectively, which are below the species delineation thresholds. No aerobic growth was observed. Cells were determined to be Gram-positive, long spindle-shaped with flagella, and non-spore-forming. The growth temperature of strain M00118^T^ ranged from 16 to 45^o^C (optimal at 30 °C). It grew at pH 6.0–8.0 (optimal at pH 7.5); no growth was observed below pH 6.0 or above pH 8.0. Strain M00118^T^ grew with 0–1 % (w/v) NaCl (optimal at 0 %), but no growth occurred above 1.0 % (w/v) NaCl. The results of the API ZYM test showed that strain M00118^T^ was positive for esterase (C4), esterase lipase (C8), leucine arylamidase, valine arylamidase, *α*-galactosidase, *β*-galactosidase, *β*-glucosidase and *α*-fucosidase, weakly positive for alkaline phosphatase, cystine arylamidase, acid phosphatase, naphthol-AS-BI-phosphohydrolase and *β*-glucuronidase, while negative for esterase (C14), trypsin, *α*-chymotrypsin, *α*-glucosidase, *N*-acetyl-*β*-glucosaminidase and *α*-mannosidase. The API 20A test results showed that strain M00118^T^ was positive for aescine iron (III) citrate *β*-glucosaccharase, *β*-glucose and cellobiose, weakly positive for d-mannose, raffinose and trehalose, and negative for gelatin, mannitol, lactose, sucrose, maltose, salicin, d-xylose, l-arabinose and glycerol. Strain M00118^T^ did not produce indole and had no catalase or urease activity. Biolog AN MicroPlates showed that its cells metabolized *N*-acetyl-d-mannosamine, amygdalin, arbutin, d-fructose, l-fructose, d-galactose, d-galacturonic acid, gentiobiose, *α*-d-glucose, glucose-6-phosphate, lactose, maltotriose, melibiose, 3-melthyl-d-glucose, methyl *α*-d-galactoside, methyl *β*-d-galactoside, methyl *β*-d-glucoside, palatinose, l-rhamnose, glyoxylic acid, *α*-ketobutyric acid, pyruvic acid, pyruvic acid methyl ester, l-serine, l-threonine, 2′-deoxy adenosine and uridine, and weakly metabolized adonitol, *α*-cyclodextrin, dextrin, glucose-1-phosphate, lactulose, sucrose, trehalose, turanose and *α*-ketovaleric acid. All Biolog AN MicroPlate results are displayed in Table S2. Strain M00118^T^ was determined to be susceptible to chloramphenicol, rifampin, penicillin, carbenicillin, ampicillin, clindamycin, amoxicillin, cefoperazone, gentamicin, tetracycline, polymyxin B, azithromycin, streptomycin, vancomycin, clarithromycin and erythromycin, and resistant to kanamycin, cefixime, ciprofloxacin and bacitracin. The major cellular fatty acids were determined as C_16 : 0_ (10.69 %), anteiso-C_15 : 0_ (25.33 %), iso-C_14 : 0_ (5.14 %), iso-C_15 : 0_ (27.70 %) and iso-C_16 : 0_ (14.92 %), and the major polar lipids were DPG, PG, unidentified GLs and unidentified PLs. The characteristics that distinguish the strain from its related taxa are presented in Table 2. Based on the phylogenetic, phylogenomic, phenotypic and chemotaxonomic results, we concluded that strain M00118^T^ represents a novel species, and the name *Acutalibacter caecimuris* sp. nov. is proposed.

**Table 2.** Differential physicochemical characteristics of the three novel strains and type strains of closely related species All strains negative for urease, catalase and indole production. +, positive; −, negative; w, weak; na, not available.

Characteristic	*A. caecimuris* ^T^	*A. intestini* ^T^	*A. muris*^T^ [1]	*N. caecimuris* ^T^	*N. timonensis*^T^ [8]
Isolation source	faeces	faeces	faeces	faeces	faeces
Gram stain	+	+	+	+	+
Spore formation	−	−	na	−	−
(°	16–45	16–45	na	16–65	28–42
(°	30	35	na	35	37
pH range for growth	6.0–8.0	6.0–8.0	na	6.0–8.0	7.0–7.5
Optimal pH for growth	7.5	7.5	na	6.5	na
(%)	0–1.0	0–0.5	na	0–1.0	0–10
(%)	0	0	na	0	na
Enzyme activity:					
Alkaline phosphatase	w	w	−	w	−
	+	+	na	w	+
Leucine arylamidase	+	+	−	+	−
Valine arylamidase	+	+	na	−	−
Naphthol-AS-BI- phosphohydrolase	w	+	na	+	+
-	+	w	+	−	+
-	+	+	+	+	−
-	−	w	w	−	+
-	−	−	−	+	−
-	+	+	+	w	−
Acid production:					
Gelatin	−	w	w	−	na
-	+	w	−	w	−
Sucrose	−	−	−	+	−
Maltose	−	+	−	−	−
Salicin	−	w	−	+	−
-	−	+	+	w	−
-	−	+	−	−	−
Cellobiose	+	w	−	−	−
-	w	w	−	−	−
Raffinose	w	−	−	−	−
-	−	+	−	−	−
-	−	−	−	w	−
Trehalose	w	−	−	−	−
Carbon source utili					
-	+	−	na	−	na
Adonitol	w	−	na	−	na
Amygdalin	+	−	na	−	−
Arbutin	+	−	na	−	−
-	w	−	na	−	na
-	−	−	na	w	na
Dextrin	w	−	+	−	na
-	+	−	na	+	na
-	+	−	na	−	−
-	+	−	na	+	−
-	+	−	na	+	na
Gentiobiose	+	−	na	+	−
-	−	−	na	w	na
-	+	−	na	−	na
Glucose-1-phosphate	w	−	na	−	na
Glucose-6-phosphate	+	w	na	w	na
*myo*-Inositol	−	+	na	−	na
	+	+	na	w	−
	+	w	na	−	na
	−	−	na	+	−
	+	−	na	+	−
	+	w	na	−	na
	+	−	na	−	na
	+	−	na	−	na
	+	−	na	−	na
Palatinose	+	−	na	−	na
Turanose	w	−	na	w	na
Fumaric acid	−	−	na	+	na
Glyoxylic acid	+	+	na	−	na
-	−	w	na	−	na
-	+	−	na	−	na
-	w	−	na	w	na
Pyruvic acid methyl ester	+	−	na	−	na
-	−	+	na	−	na
Glycyl-l-methionine	−	−	na	+	na
-	−	−	na	+	na
-	−	−	na	w	na
-	+	−	na	−	na
-	+	−	na	w	na
-	−	−	na	+	na
- +	−	−	na	+	na
′-	+	−	na	+	na
Uridine	+	−	na	−	na
)	_16 : 015 : 014 : 015 : 016 : 0_	_16 : 016 : 015 : 015 : 016 : 0_	_16 : 018 : 115 : 015 : 016 : 0_	_16 : 015 : 014 : 015 : 0_	_14 : 016 : 016 : 015 : 015 : 0_
Polar lipids*	DPG, PG, GL1, GL2, GL3, GL4, PL1, GL2	DPG, PG, GL1, GL2, GL3, GL4, GL5, PL1, PL2, PL3	na	DPG, PG, GL1, GL2, GL3, GL4, GL5, PL1, PL2, PL3, PL4, PL5, PL6, PL7	na
Major respiratory quinone*	MK-6	MK-6	na	MK-6	na

*DPG, diphosphatidylglycerol; PG, phosphatidylglycerol; GL, glycolipids; PL, phospholipid; MK-6, menaquinone 6.

#### Strain M00204^T^

Phylogenetic analyses showed that strain M00204^T^ clustered with members of the genus *Acutalibacter* of the family *Oscillospiraceae* and was closest to *A. muris* KB18^T^ (96.64 %, 16S rRNA gene sequence identity). The genome size of M00204^T^ was 4 293 150 bp, and the G+C content was determined to be 53.44 mol%. kegg annotation yielded 143 genes related to carbohydrate metabolism, 105 genes related to amino acid metabolism, 58 genes related to metabolism of cofactors and vitamins, 74 genes related to nucleotide metabolism, 67 genes related to energy metabolism, 36 genes related to lipid metabolism, 35 genes related to glycan biosynthesis and metabolism, 23 genes related to biosynthesis of other secondary metabolites, 12 genes related to metabolism of terpenoids and polyketides, and 11 genes related to xenobiotic biodegradation and metabolism. The ANI and the dDDH values between the genomes of strain M00204^T^ and *A. muris* KB18^T^ were 77.05 and 29.40 %, respectively, which are below the species thresholds of 95 [33] and 70 % [32], respectively. No aerobic growth was observed. Cells were Gram-stain-positive, rod-shaped with flagella, anaerobic, and non-spore-forming. Growth occurred at 16–45^o^C (optimal at 35 °C) and at pH 6.0–8.0 (optimal at pH 7.5; no growth was observed below pH 6.0 or above 8.0). Strain M00204^T^ grew with 0–0.5 % (w/v) NaCl (optimal at 0 %); no growth occurred above 0.5 % (w/v) NaCl. The API ZYM test results showed that strain M00204^T^ was positive for esterase (C4), esterase lipase (C8), leucine arylamidase, valine arylamidase, acid phosphatase, naphthol-AS-BI-phosphohydrolase, *α*-galactosidase, *β*-glucosidase and *α*-fucosidase, weakly positive for alkaline phosphatase, cystine arylamidase, *β*-galactosidase and *α*-glucosidase, while negative for esterase (C14), trypsin, *α*-chymotrypsin, *β*-glucuronidase, *N*-acetyl-*β*-glucosaminidase and *α*-mannosidase. The results of the API 20A tests showed that strain M00204^T^ was positive for aescine iron (III) citrate *β*-glucosaccharase, maltose, d-xylose, l-arabinose and d-sorbitol, weakly positive for gelatin, *β*-glucose, salicin, cellobiose and d-mannose, and negative for gelatin, mannitol, lactose, sucrose, glycerol, melezitose, raffinose, l-rhamnose and trehalose. All Biolog AN MicroPlate results are displayed in Table S2. Strain M00204^T^ did not produce indole and had no catalase or urease activity. Biolog AN MicroPlates showed that its cells metabolized *myo*-inositol, lactose, glyoxylic acid and l-alanyl-l-threonine, and weakly metabolized glucose-6-phosphate, lactulose, maltotriose, 3-methyl-d-glucose, sucrose, trehalose and *β*-hydroxybutyric acid. Strain M00204^T^ was susceptible to kanamycin, chloramphenicol, rifampin, penicillin, carbenicillin, ampicillin, amoxicillin, gentamicin, tetracycline, azithromycin, streptomycin, vancomycin, clarithromycin and erythromycin and resistant to cefixime, clindamycin, cefoperazone, ciprofloxacin, polymyxin B and bacitracin. The major cellular fatty acids were determined as C_16 : 0_ (7.16 %), C_16 : 1_ 2-OH (5.17 %), anteiso-C_15 : 0_ (26.01 %), iso-C_15 : 0_ (33.05 %) and iso-C_16 : 0_ (11.40 %), and the major polar lipids were DPG, PG, unidentified GLs and unidentified PL. The characteristics that differentiate this strain from its related taxa are presented in Table 2. Based on the phylogenetic, phylogenomic, phenotypic and chemotaxonomic results, we concluded that strain M00204^T^ represents a novel species in the genus *Acutalibacter*, and the name *Acutalibacter intestini* sp. nov. is proposed.

#### Strain M00184^T^

Phylogenetic analyses revealed that strain M00184^T^ clustered within the genus of *Neglectibacter* of the family *Oscillospiraceae*, and was closest to *N. timonensis* SN17^T^ (96.54 %, 16S rRNA gene sequence identity). The genome size of M00184^T^ was 3 089 163 bp and the G+C content was determined to be 52.90 mol%. kegg annotation of the genome yielded 164 genes related to carbohydrate metabolism, 133 genes related to amino acid metabolism, 69 genes related to cofactors and vitamins, 67 genes related to nucleotide metabolism, 72 genes related to energy metabolism, 36 genes related to lipid metabolism, 36 genes related to glycan biosynthesis and metabolism, 36 genes related to biosynthesis of other secondary metabolites, 15 genes related to metabolism of terpenoids and polyketides, and 13 genes related to xenobiotic biodegradation and metabolism. The ANI and the dDDH values between the genomes of strains M00184^T^ and *N. timonensis* SN17^T^ were 72.13 and 21.20 %, respectively, which are below the species thresholds of 95 [33] and 70 % [32], respectively. No aerobic growth was observed. Cells were Gram-stain-positive, short rod-shaped and non-spore-forming. The growth temperature of strain M00184^T^ ranged from 16 to 45^o^C (optimal at 35 °C). Strain M00184^T^ grew at pH 6.0–8.0 (optimal at pH 6.5; no growth was observed below pH 6.0 or above pH 8.0). Strain M00184^T^ grew with 0–1 % (w/v) NaCl (optimal at 0 %), but no growth occurred above 1.0 % (w/v) NaCl. The results of the API ZYM tests showed that strain M00184^T^ was positive for esterase (C4), leucine arylamidase, acid phosphatase, naphthol-AS-BI-phosphohydrolase, *α*-galactosidase, *β*-galactosidase, *β*-glucuronidase, *β*-glucosidase and *N*-acetyl-β-glucosaminidase, weakly positive for alkaline phosphatase, esterase lipase (C8) and *α*-fucosidase, while negative for esterase (C14), valine arylamidase, cystine arylamidase, trypsin, *α*-chymotrypsin, *α*-glucosidase and *α*-mannosidase. The API 20A test results showed that strain M00184^T^ was positive for aescine iron (III) citrate *β*-glucosaccharase, sucrose and salicin, weakly positive for *β*-glucose, d-xylose and l-rhamnose, and negative for gelatin, mannitol, lactose, maltose, l-arabinose, glycerol, cellobiose, d-mannose, melezitose, raffinose, d-sorbitol and trehalose. Strain M00184^T^ did not produce indole and had no catalase or urease activity. Biolog AN MicroPlates showed that cells metabolized d-fructose, d-galactose, d-galacturonic acid, gentiobiose, *α*-d-glucose, d-mannitol, melezitose, melibiose, fumaric acid, glycyl-l-methionine, l-methionine, l-valine and l-valine +l-aspartic acid and 2′-deoxyadenosine, and weakly metabolized *β*-cyclodextrin, d-glucosaminic acid, glucose-6-phosphate, lactose, turanose, *α*-ketovaleric acid, l-phenylalanine and l-threonine. All Biolog AN MicroPlate results are displayed in Table S2. Strain M00184^T^ was susceptible to chloramphenicol, rifampin, penicillin, carbenicillin, ampicillin, amoxicillin, cefoperazone, gentamicin, tetracycline, azithromycin, streptomycin, vancomycin, clarithromycin and erythromycin, and resistant to kanamycin, cefixime, clindamycin, ciprofloxacin, polymyxin B and bacitracin. The major cellular fatty acids were determined as C_16 : 0_ (5.55 %), anteiso-C_15 : 0_ (30.11 %), iso-C_14 : 0_ (7.69 %) and iso-C_15 : 0_ (40.20 %), and the major polar lipids were DPG, PG, unidentified GLs and unidentified PL. The characteristics that differentiate this strain from related taxa are presented in Table 2. Based on the phylogenetic, phenotypic and chemotaxonomic results, we concluded that strain M00184^T^ represents a novel species, and the name *Neglectibacter caecimuris* sp. nov. is proposed.

## Description of *Acutalibacter caecimuris* sp. nov.

*Acutalibacter caecimuris* (cae.ci.mu’ris. N. L. neut. n. *caecum,* caecum; L. masc., n. *mus*, a mouse; N. L. gen. n. *caecimuris*, from the caecum of a mouse).

Cells are Gram-positive, strictly anaerobic, non-spore-forming, rod-shaped with flagella, 1.0–2.5 µm long and around 0.5 µm wide, depending on the growth stage. Forms smooth, irregular, cloudy white colonies with rounded edges on mGAM agar plates after incubation at 37 °C for 3 days. Growth occurs at 16–45 °C (optimal at 30 °C) and pH 6–8 (optimal at pH 7.5). Grows in 0–1.0 % NaCl (w/v; optimal at 0 %). Does not use tryptophan to produce indole, and has no urease or catalase activity. Positive for esterase (C4), esterase lipase (C8), leucine arylamidase, valine arylamidase, *α*-galactosidase, *β*-galactosidase, *β*-glucosidase and *α*-fucosidase. Acid production due to utilization of aescine iron (III) citrate *β*-glucosaccharise, *β*-glucose and cellobiose. Strain M00118^T^ can utilize *N*-acetyl-d-mannosamine, amygdalin, arbutin, cellobiose, d-fructose, l-fucose, d-galactose, d-galacturonic acid, gentiobiose, *α*-d-glucose, glucose-6-phosphate, lactose, d-maltotriose, melibiose, 3-melthyl-d-glucose, methyl *α*-d-galactoside, methyl *β*-d-galactoside, methyl *β*-d-glucoside, palatinose, glyoxylic acid, *α*-ketobutyric acid, pyruvic acid, pyruvic acid methyl ester, l-serine, l-threonine, 2′-deoxy adenosine and uridine. The major polar lipids are DPG, PG, GL and PL. The predominant cellular fatty acids are C_16 : 0_, anteiso-C_15 : 0_, iso-C_14 : 0_, iso-C_15 : 0_ and iso-C_16 : 0_. The major respiratory quinone is MK6.

The type strain, M00118^T^ (=CGMCC 1.18042^T^=KCTC 25739^T^), was isolated from the caeca of a male C57BL/6J mouse in China. The DNA G+C content of the type strain is 56.67 mol%. GenBank accessions for the whole genome and 16S rRNA gene sequences are JAWXXJ000000000 and OR791045, respectively.

## Description of *Acutalibacter intestini* sp. nov.

*Acutalibacter intestini* (in.tes’ti.ni. N. L. gen. n. *intestini,* of the gut, referring to the isolation origin of the type strain).

Cells are Gram-positive, strictly anaerobic, non-spore-forming, rod-shaped with flagella, 5.5–8.0 µm long and 0.5–1.0 µm wide, depending on their growth stage. Forms creamy white colonies with smooth surfaces and irregular round edges on mGAM after incubation at 37 °C for 3 days. Growth occurs at 16–45 °C (optimal at 35 °C) and pH 6–8 (optimal at pH 7.5). Growth in 0–0.5 % NaCl (w/v, optimal at 0). Does not use tryptophan to produce indole, and has no urease or catalase activity. Positive for esterase (C4), esterase lipase (C8), leucine arylamidase, valine arylamidase, acid phosphatase, naphthol-AS-BI-phosphohydrolase, *α*-galactosidase, *β*-glucosidase and *α*-fucosidase. Acid production due to utilization of aescine iron (III) citrate *β*-glucosaccharase, maltose, d-xylose, l-arabinose and d-sorbitol. Strain M00204^T^ can utilize *myo*-inositol, lactose, glyoxylic acid and l-alanyl-l-threonine. The major polar lipids are DPG, PG, GL and PL. The major cellular fatty acids are C_16 : 0_, C_16 : 0_ 2-OH, anteiso-C_15 : 0_, iso-C_15 : 0_ and iso-C_16 : 0_. The major respiratory quinone is MK6.

The type strain, M00204^T^ (=CGMCC 1.18044^T^=KCTC 25741^T^), was isolated from the caeca of a male C57BL/6J mouse in China. The DNA G+C content of the type strain is 53.44 mol%. GenBank accession numbers for the whole genome and 16S rRNA gene sequences of strain M00204^T^ are JAWXXL000000000 and OR791047, respectively.

## Description of *Neglectibacter caecimuris* sp. nov.

*Neglectibacter caecimuris* (cae.ci.mu’ris. N. L. neut. n. *caecum* caecum; L. masc., n. *mus*, a mouse; N. L. gen. n. *caecimuris*, from the caecum of a mouse).

Cells are anaerobic, Gram-positive, non-spore-forming, short rod-shaped with no flagella, 1.5–2.3 µm long and 0.6–1.5 µm wide. They form smooth, uniform, glossy white colonies with regular round edges on mGAM agar plates after incubation at 37 °C for 1 week. Growth occurs at 16–65 °C (optimal at 35 °C) and pH 6–8 (optimal at pH 6.5). Growth in 0–1 % NaCl (w/v; optimal at 0 %). Does not use tryptophan to produce indole and has no urease or catalase activity. Positive for esterase (C4), leucine arylamidase, acid phosphatase, naphthol-AS-BI-phosphohydrolase, *α*-galactosidase, *β*-galactosidase, *β*-glucuronidase, *β*-glucosidase and *N*-acetyl-*β*-glucosaminidase. Acid production due to utilization of aescine iron (III) citrate *β*-glucosaccharase, sucrose and salicin. Strain M0184^T^ can utilize d-fructose, d-galactose, d-galacturonic acid, gentiobiose, lactose, melezitose, melibiose, fumaric acid, glycyl-l-methionine, l-methionine, l-valine, l-valine +l-aspartic acid, 2′-deoxyadenosine. The major polar lipids are DPG, PG, GL and PL. The major cellular fatty acids are C_16 : 0_, anteiso-C_15 : 0_, iso-C_14 : 0_ and iso-C_15 : 0_. The major respiratory quinone is MK6.

The type strain, M00184^T^ (=CGMCC 1.18043^T^=KCTC 25740^T^), was isolated from the caeca of a C57BL/6J mouse in China. The DNA G+C content of the type strain is 52.90 mol%. GenBank accession numbers for the whole genome and the 16S rRNA gene sequences of strain M00184^T^ are JAWXXK000000000 and OR791046, respectively.

## supplementary material

10.1099/ijsem.0.006449 Uncited Supplementary Material 1.

## Abbreviations

ANI: average nucleotide identity
dDDH: digital DNA–DNA hybridization
mGAM: modified Gifu anaerobic medium
ML: maximum likelihood
MP: maximum-parsimony
NJ: neighbour-joining
