# Supplementary material for: Acutalibacter caecimuris sp. nov., Acutalibacter intestini sp. nov. and Neglectibacter caecimuris sp. nov., three novel species of the family Oscillospiraceae isolated from caecal contents of C57BL/6J mice
Source: Int J Syst Evol Microbiol. 2024 Jul 12;74(7):006449. doi: 10.1099/ijsem.0.006449 (PMC11316587; doi:10.1099/ijsem.0.006449)
Supplement: Uncited Supplementary Material 1. [file ijsem-74-06449-s001.pdf]

# *Acutalibacter caecimuris* sp. nov., *Acutalibacter intestini* sp. nov., *and Neglectibacter caecimuris* sp. nov., three novel species of the family *Oscillospiraceae* isolated from cecal contents of C57BL/6J mice

Jia-Hui He[1,2], Chang-Yu Wang[4], Rashidin Abdugheni[5], Xue Ni[3], Chang Liu[3], Ming-Xia Bi[3*], Shuang-Jiang Liu[2,3*]

[1]College of Veterinary Medicine, Shanxi Agricultural University (Shanxi Academy of Agricultural Sciences), Jinzhong, 030801, China.

[2]State Key Laboratory of Microbial Resources, Institute of Microbiology, Chinese Academy of Sciences, Beijing, 100101, P. R. China

[3]State Key Laboratory of Microbial Technology, Shandong University, Qingdao, 266237, P. R. China.

[4]School of Life Sciences, Division of Life Sciences and Medicine, University of Science and Technology of China, Hefei, 230001, China.

[5]State Key Laboratory of Desert and Oasis Ecology, Key Laboratory of Ecological Safety and Sustainable Development in Arid Lands, Xinjiang Institute of Ecology and Geography, Chinese Academy of Sciences, Urumqi, 830011, China.

*Corresponding authors:

Shuang-Jiang Liu (liusj@im.ac.cn)

Ming-Xia Bi (bimx@sdu.edu.cn)

Postal address: State Key Laboratory of Microbial Technology, Shandong University, Binhai Road No. 72, Jimo District, Qingdao 266237, China

Telephone number: + 0532-58630001

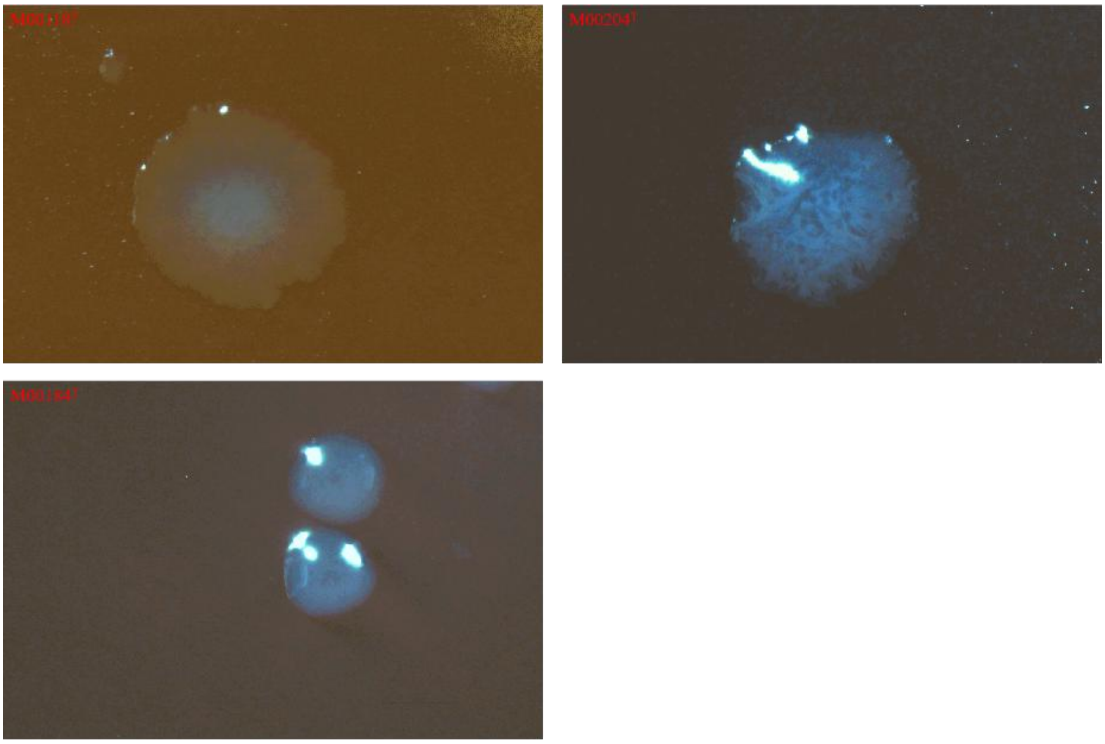

**Figure S1. Colony morphology of three strains.**

**M00118$^{T}$:** After incubation at 37°C for 3 days anaerobically, smooth, translucent, omelette-like surface with irregular edges and not glistening colonies appeared on the mGAM agar plates; **M00204$^{T}$:** After incubation at 37°C for 7 days anaerobically, creamy white, smooth, opaque, raised and with irregular edges, glossy colonies appeared on the mGAM agar plates; **M00184$^{T}$:** After incubation at 37°C for 3 days anaerobically, white, smooth, opaque, rounded, raised, neatly edged and shiny colonies appeared on the mGAM agar plates.

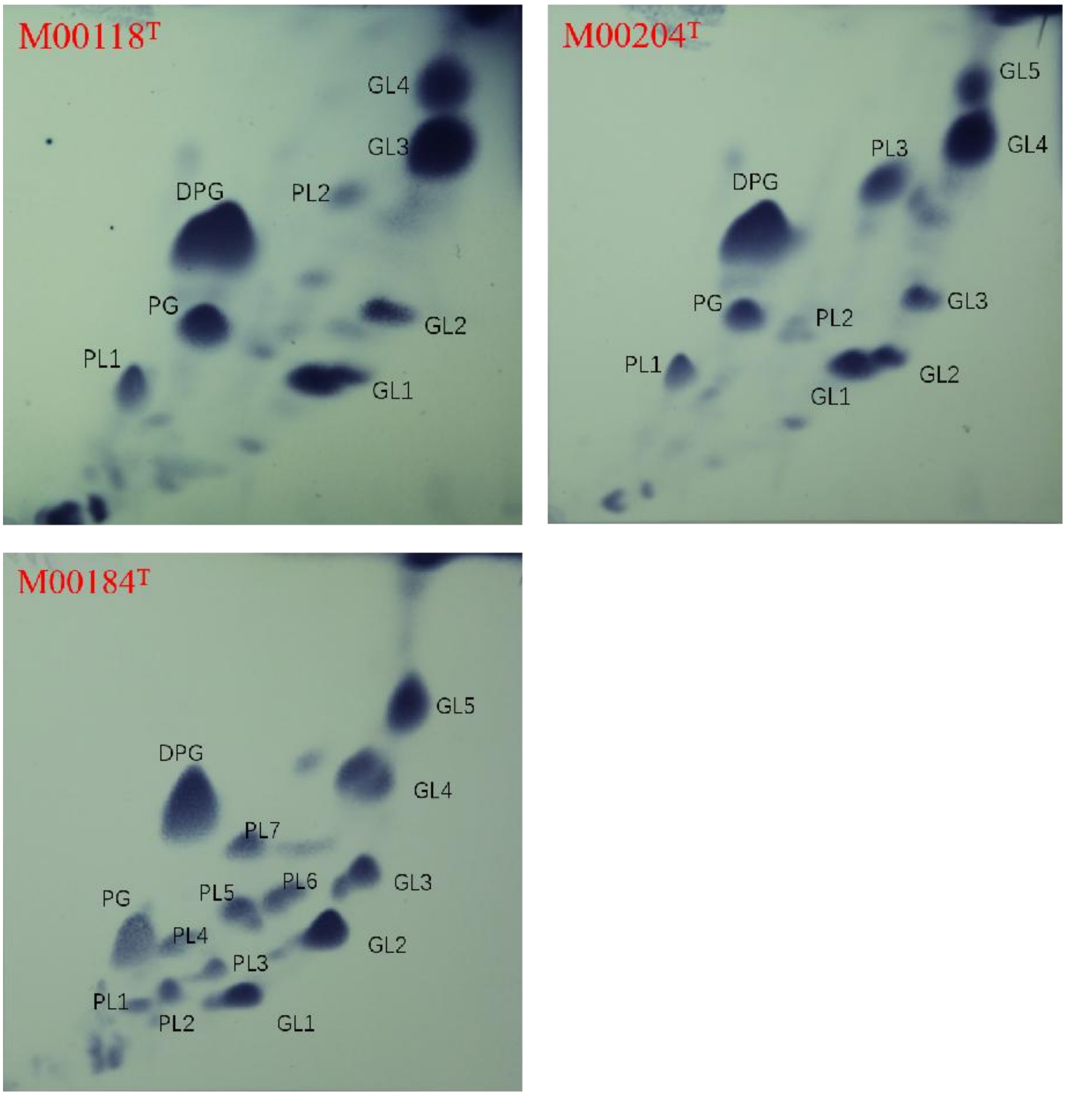


**Figure S2. Polar lipid profiles after separation by two-dimensional thin layer chromatography of the three strains.**

DPG, diphosphatidylglycerol; PG, phosphatidylglycerol; PL, phospholipids; GL, glycolipids.

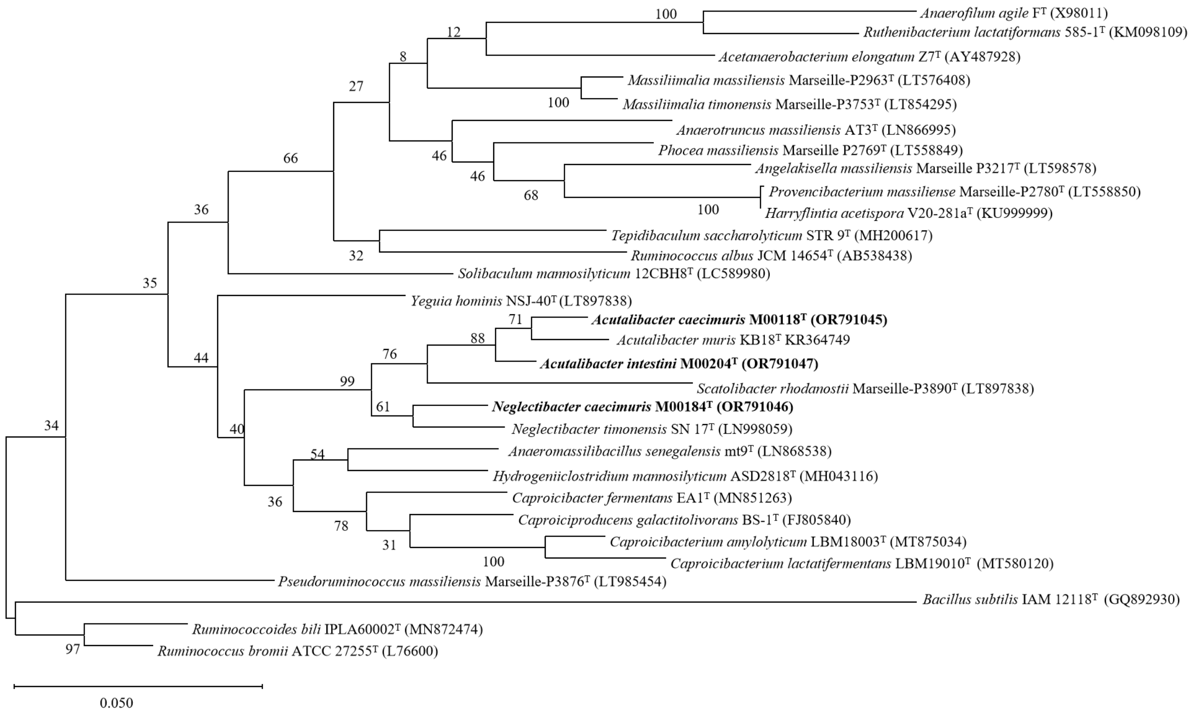


a

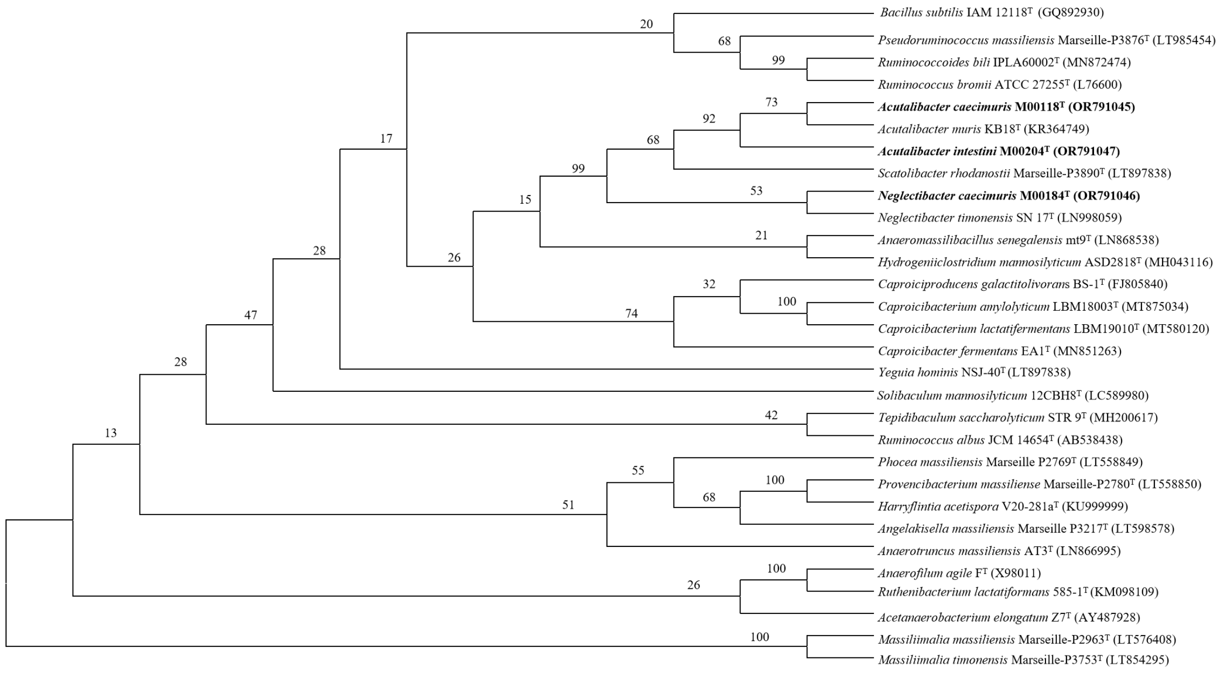


b

**Figure S3. Phylogenetic tree based on the 16S rRNA gene constructed with Maximum Likelihood (ML) (a) and Maximum Parsimony (MP) (b) method**. Bar, 0.05 substitutions per nucleotide position.

**Table S1. Cellular fatty acid composition (%) of the three strains and type strains of closely related species.**

TR, trace amounts < 1 %; ND, not detected; NA: not available.

| **Fatty Acids** | ***A. caecimuris* M00118$^T$** | ***A. intestini* M00204$^T$** | ***A. muri* KB18$^T$ [1]** | ***N. caecimuris* M00184$^T$** | ***N. timonensis* SN17$^T$ [2]** |
|---|---|---|---|---|---|
| $C_{14:0}$ | 4.41 | 3.02 | NA | 2.58 | **10** |
| $C_{16:0}$ | **10.69** | **7.16** | **22.1** | **5.55** | **31.3** |
| $C_{18:0}$ | 2.72 | 3.73 | NA | 2.69 | NA |
| $C_{13:1}$ at 12-13 | ND | ND | NA | 1.21 | NA |
| $C_{16:1}$ 2-OH | TR | **5.17** | NA | TR | NA |
| $C_{16:0}$ N-alcohol | ND | ND | NA | TR | NA |
| $C_{16:0}$-aldehyde | ND | ND | NA | ND | **18.3** |
| $C_{18:1}$ $\omega$9c | 1.25 | 2.19 | **12.2** | 1.56 | NA |
| anteiso- $C_{15:0}$ | **25.33** | **26.01** | **9.8** | **30.11** | **12.3** |
| anteiso- $C_{17:0}$ | 2.69 | 1.95 | NA | ND | NA |
| iso-$C_{13:0}$ | ND | ND | NA | TR | NA |
| iso-$C_{14:0}$ | **5.14** | 3.76 | NA | **7.69** | NA |
| iso-$C_{15:0}$ | **27.70** | **33.05** | **16.8** | **40.20** | **11.1** |
| iso-$C_{16:0}$ | **14.92** | **11.40** | NA | 2.68 | NA |
| iso-$C_{17:0}$ | 3.08 | 1.20 | NA | ND | NA |
| iso-$C_{12:0}$ 3-OH | ND | ND | NA | 1.20 | NA |
| iso-$C_{16:0}$ DMA | ND | ND | **21.7** | ND | NA |
| Summed Feature: * | | | | | |
| 1 | TR | 1.37 | NA | 2.02 | NA |
| 5 | TR | ND | NA | ND | NA |

*Summed Features are fatty acids that cannot be resolved reliably from another fatty acid using the chromatographic conditions chosen. The MIDI system groups these fatty acids together as one feature with a single percentage of the total. *Summed Feature1 contains iso-$C_{15:1}$-H and/ or $C_{13:0}$ 3-OH; Summed Feature 5 contains ante-$C_{18:0}$ and/ or $C_{18:0}$ $\omega$6,9c.

**Table S2. Biolog AN data of the three strains.**

+, positive; -, negative; W, weak.

| **Carbon source utilisation** | ***A. caecimuris* M00118$^T$** | ***A. intestini* M00204$^T$** | ***N. caecimuris* M00184$^T$** |
|---|---|---|---|
| Water | - | - | - |
| N-acetyl-D-galactosamine | - | - | - |
| N-acetyl-D-mannosamine | + | - | - |
| N-acetyl-*β*-D-mannosamine | - | - | - |
| Adonitol | W | - | - |
| Amygdalin | + | - | - |
| D-arabitol | - | - | - |
| Arbutin | + | - | - |
| D-cellobiose | + | W | - |
| *α*-cyclodextrin | W | - | - |
| *β*-cyclodextrin | - | - | W |
| Dextrin | W | - | - |
| Dulcitol | - | - | - |
| i-erythritol | - | - | - |
| D-fructose | + | - | + |
| L-fucose | + | - | - |
| D-galactose | + | - | + |
| D-galacturonic acid | + | - | + |
| Gentiobiose | + | - | + |
| D-gluconic acid | - | - | - |
| D-glucosaminic acid | - | - | W |
| *α*-D-glucose | + | - | - |
| Glucose-1-phosphate | W | - | - |
| Glucose-6-phosphate | + | W | W |
| Glycerol | - | - | - |
| D, L-*α*-glycerol phosphate | - | - | - |
| m-Inositol | - | + | - |
| *α*-D-lactose | + | + | W |
| Lactulose | W | W | - |
| Maltose | - | + | - |
| D-maltotriose | + | W | - |
| D-mannitol | - | - | - |
| D-mannose | W | W | - |
| D-melezitose | - | - | + |
| D-melibiose | + | - | + |
| 3-melthyl-D-glucose | + | W | - |
| *α*-methyl-D-galactoside | + | - | - |
| *β*-methyl-D-galactoside | + | - | - |

| | | | |
|---|---|---|---|
| α-methyl-D-glucoside | - | - | - |
| β-methyl-D-glucoside | + | - | - |
| Palatinose | + | - | - |
| D-raffinose | - | - | - |
| L-rhamnose | - | - | W |
| Salicin | - | W | + |
| D-sorbitol | - | + | - |
| Stachyose | - | - | - |
| Sucrose | W | W | - |
| D-trehalose | W | W | - |
| Turanose | W | - | W |
| Acetic acid | - | - | - |
| Formic acid | - | - | - |
| Fumaric acid | - | - | + |
| Glyoxylic acid | + | + | - |
| α-Hydroxybutyric acid | - | - | - |
| β-Hydroxybutyric acid | - | W | - |
| Itaconic | - | - | - |
| α-ketobutyric acid | + | - | - |
| α-ketovaleric acid | W | - | W |
| D, L-lactic acid | - | - | - |
| L-lactic acid | - | - | - |
| D-lactic acid methyl ester | - | - | - |
| D-malic acid | - | - | - |
| L- malic acid | - | - | - |
| Propionic acid | - | - | - |
| Pyruvic acid | - | - | - |
| Pyruvic acid methyl ester | + | - | - |
| D-saccharic acid | - | - | - |
| Succinamic acid | - | - | - |
| Succinic acid | - | - | - |
| Succinic acid mono-methyl ester | - | - | - |
| m-tartaric acid | - | - | - |
| Urocanic acid | - | - | - |
| L-alaninamide | - | - | - |
| L-alaninamide | - | - | - |
| L-alanyl-L-glutamine | - | - | - |
| L-alanyl-L-histidin | - | - | - |
| L-alanyl-L-threonine | - | + | - |
| L-asparagine | - | - | - |
| L-glutamic acid | - | - | - |
| L-glutamine | - | - | - |
| Glycyl-L-aspartic acid | - | - | - |

| | | | |
|---|---|---|---|
| Glycyl-L-glutamine | - | - | - |
| Glycyl-L-methionine | - | - | + |
| Glycyl-L-proline | - | - | - |
| L-methionine | - | - | + |
| L-phenylalanine | - | - | W |
| L-serine | + | - | - |
| L-threonine | + | - | W |
| L-valine | - | - | + |
| L-valine + L-aspartic acid | - | - | + |
| 2’-Deoxyadenosine | + | - | + |
| Inosine | - | - | - |
| Thymidine | - | - | - |
| Uridine | + | - | - |
| Thymidine-5’-monophosphate | - | - | - |
| Uridine-5’-monophosphate | - | - | - |

## References


1. **Lagkouvardos I, Pukall R, Abt B, Foesel BU, Meier-Kolthoff JP et al.** The Mouse Intestinal Bacterial Collection (miBC) provides host-specific insight into cultured diversity and functional potential of the gut microbiota. *Nature Microbiology* 2016;1(10):16131.
2. **Zgheib R, Ibrahim A, Anani H, Ndongo S, Bilen M et al.** *Neglectibacter timonensis* gen. nov., sp. nov. and *Scatolibacter rhodanostii* gen. nov., sp. nov., two anaerobic bacteria isolated from human stool samples. *Archives of Microbiology* 2021;204(1):45.
